# A Study on Prevalence and Characterization of *Bacillus cereus* in Ready-to-Eat Foods in China

**DOI:** 10.3389/fmicb.2019.03043

**Published:** 2020-01-15

**Authors:** Shubo Yu, Pengfei Yu, Juan Wang, Chun Li, Hui Guo, Chengcheng Liu, Li Kong, Leyi Yu, Shi Wu, Tao Lei, Moutong Chen, Haiyan Zeng, Rui Pang, Youxiong Zhang, Xianhu Wei, Jumei Zhang, Qingping Wu, Yu Ding

**Affiliations:** ^1^State Key Laboratory of Applied Microbiology Southern China, Guangdong Provincial Key Laboratory of Microbial Culture Collection and Application, Guangdong Open Laboratory of Applied Microbiology, Guangdong Institute of Microbiology, Guangdong Academy of Sciences, Guangzhou, China; ^2^Department of Food Science and Technology, Institute of Food Safety and Nutrition, Jinan University, Guangzhou, China; ^3^College of Food Science, South China Agricultural University, Guangzhou, China

**Keywords:** *Bacillus cereus*, ready-to-eat food, risk assessment, virulence genes, antibiotic resistance, genetic polymorphism

## Abstract

*Bacillus cereus* is widely distributed in different food products and can cause a variety of symptoms associated with food poisoning. Since ready-to-eat (RTE) foods are not commonly sterilized by heat treatment before consumption, *B. cereus* contamination may cause severe food safety problems. In this study, we investigated the prevalence of *B. cereus* in RTE food samples from different regions of China and evaluated the levels of bacterial contamination, antibiotic resistance, virulence gene distribution, and genetic polymorphisms of these isolates. Of the tested retail RTE foods, 35% were positive for *B. cereus*, with 39 and 83% of the isolated strains harboring the enterotoxin-encoding *hblACD* and *nheABC* gene clusters, respectively. The *entFM* gene was detected in all *B*. *cereus* strains. The *cytK* gene was present in 68% of isolates, but only 7% harbored the emetic toxin-encoding gene *cesB*. Antimicrobial susceptibility testing revealed that the majority of the isolates were resistant not only to most β-lactam antibiotics, but also to rifamycin. Multilocus sequence typing (MLST) revealed that the 368 isolates belonged to 192 different sequence types (STs) including 93 new STs, the most prevalent of which was ST26. Collectively, our study indicates the prevalence, bacterial contamination levels, and biological characteristics of *B. cereus* isolated from RTE foods in China and demonstrates the potential hazards of *B. cereus* in RTE foods.

## Introduction

Ready-to-eat (RTE) foods, such as cooked meats and poultry, cold vegetable dishes in sauce, cold noodles, and fried rice, are very popular as they are intended for direct consumption. Although they are very convenient for consumers, RTE foods have been shown to be frequently contaminated with pathogenic bacteria such as *Bacillus cereus*, *Listeria monocytogenes*, and *Staphylococcus aureus* (Batchoun et al., 2011; Hwang and Park, 2015; Wu et al., 2016; Yang et al., 2016).

*Bacillus cereus* is a gram-positive bacterium that causes foodborne diseases and is widespread in nature and foods (Marrollo, 2016). *B. cereus* has been isolated from a variety of foods, particularly RTE foods such as cooked rice and mixed salad (Park et al., 2009; Batchoun et al., 2011; Rahimi et al., 2013; Tewari et al., 2015; Gao et al., 2018; Yu et al., 2019). *B. cereus* can cause food poisoning even at very low doses, with more than 10^3^ *B. cereus* g^–1^ considered unsafe for consumption (Granum and Lund, 1997). Despite safety precautions, numerous food poisoning incidents caused by *B. cereus* have been reported recently in Spain (Doménech-Sánchez et al., 2011), Belgium (Delbrassinne et al., 2015), Argentina (Lopez et al., 2015), Australia (Sloan-Gardner et al., 2014), England (Nicholls et al., 2016), Austria (Schmid et al., 2016), and France (Glasset et al., 2016).

*Bacillus cereus* produces a range of virulence factors and can enter the gastrointestinal tract via ingestion, where it causes diarrhea and vomiting (Jensen et al., 2003; Stenfors Arnesen et al., 2008; Song et al., 2019). Diarrhea is associated with four different enterotoxins, the hemolysin BL (HBL, encoded by *hblA*, *hblC*, and *hblD*), non-hemolytic enterotoxin (NHE, encoded by *nheA*, *nheB*, *nheC*), enterotoxin FM (EntFM, encoded by *entFM*) and the cytotoxin K (CytK, encoded by *cytK*) (Beecher et al., 1995; Lund and Granum, 1996; Granum et al., 1999; Lund et al., 2000; Bonerba et al., 2010; Tran et al., 2010; Berthold-Pluta et al., 2019). HBL and NHE are both tripartite toxins (Berthold-Pluta et al., 2019). CytK belongs to a member of the family of β-barrel pore forming toxins that can cause serious food poisoning, skin necrosis, hemolysis, and even death (Lund et al., 2000). EntFM is related to cell wall peptidases (CWPs) which can cause diseases such as diarrhea (Tran et al., 2010). Whilst vomiting, or emesis, is induced by a small, heat and acid stable cyclic dodecadepsipeptide ([D-*O*Leu-D-Ala-L-*O*-Val-L-Val]3) toxin known as cereulide that is synthesized by non-ribosomal peptide synthetases encoded by *ces* genes (Ehling-Schulz et al., 2005, 2015). Besides food poisoning, *B. cereus* is also associated with serious infections such as pneumonia, bacteremia, endophthalmitis, necrotizing fasciitis, osteomyelitis, and endocarditis (Bottone, 2010; Rishi et al., 2013; Ikeda et al., 2015).

Antibiotic treatment is still the main method for treating bacterial infections, including those caused by *B. cereus*; however, the extensive use of antimicrobials has led to the emergence of antibiotic-resistant strains, including those resistant to multiple antibiotics, which can cause routine treatments to fail (Friedman et al., 2016). Thus, determining the antibiotic resistance profile of *B. cereus* is important for informing drug selection for treatment regimens.

The contamination of RTE foods by pathogenic bacteria such as *B. cereus* is a major food safety concern; thus, it is necessary to monitor and characterize *B. cereus* contamination in RTE foods. This study investigated the potential pathogenicity, contamination levels, molecular characteristics, and antibiotic resistance profiles of *B. cereus* isolated from RTE foods in China, providing important information about the prevalence of *B. cereus* in RTE foods.

## Materials and Methods

### Sample Collection

A total of 860 RTE food samples were collected from retail markets and supermarkets in 39 major Chinese cities (Supplementary Figure S1) between 2011 and 2016 according to the general guidelines of the National Food Safety Standard in Sample Collection (The Hygiene Ministry of China, 2010). The samples included cooked meat (656 samples), cold vegetable dishes in sauce (85 samples), and rice/noodles (119 samples). All samples were placed in separate sterile bags, transferred to the laboratory on ice within 2 days, and kept below 4°C.

### Qualitative and Quantitative Detection of *B. cereus*

*Bacillus cereus* was qualitatively and quantitatively detected according to the bacteriological analytical manuals of the U.S. Food and Drug Administration and the National Food Safety Standard of China (The Hygiene Ministry of China, 2003; Tallent et al., 2012). In brief, 25 g samples were randomly collected from each RTE food sample and put into sterile blender jar with 225 mL Trypticase Soy Broth (TSB) with polymyxin (Huankai, Guangzhou, China), then blended for 2 min at high speed (10,000 to 12,000 rpm). Homogenates were incubated 48 ± 2 h at 30 ± 2°C. Afterward, a loop of the resulting cultures was streaked onto mannitol egg yolk polymyxin agar plates (MYP) (Huankai), which were incubated 24 h at 30°C. Single colonies were then streaked onto chromogenic *B. cereus* agar plates (Huankai). Different presumptive colonies from the chromogenic *B. cereus* agar plates were picked for further biochemical characterization using a *B. cereus* biochemistry assessor (Huankai) to identify authentic colonies. The most probable number (MPN) method was used for the quantitative detection of *B. cereus*. A three-tube MPN series was inoculated into TSB with polymyxin, using 1 mL inoculums of 10^–1^, 10^–2^, and 10^–3^ dilutions of each sample, with three tubes at each dilution. The tubes were incubated for 48 ± 2 h at 30 ± 2°C and observed for turbid growth typical of *B*. *cereus*. The cultures from turbid, positive tubes were streaked onto MYP agar plates and incubated for 24 h at 30°C. One or more pink, lecithin-positive colonies were selected from each MYP agar plate and further confirmed on chromogenic *B. cereus* agar plates. The number of tubes confirmed as positive for *B. cereus* was used to calculate the MPN of *B. cereus* per g (mL) sample, expressed as MPN/g (mL) using the MPN table.

### Virulence Gene Distribution

Genomic DNA was extracted using a genomic DNA extraction kit for gram-positive bacteria (Magen, Guangzhou, China) according to the manufacturer’s instructions. Different virulence genes, including *hblA*, *hblC*, *hblD*, *nheA*, *nheB*, *nheC*, *entFM*, *cytK*, and *cesB*, were detected by PCR using the primers listed in Supplementary Table S1 with previously described thermal profiles (Hansen and Hendriksen, 2001; Ehling-Schulz et al., 2005; Oltuszak-Walczak and Walczak, 2013; Forghani et al., 2014; Yu et al., 2019).

### ERIC-PCR

The ERIC-PCR were conducted by using the primers listed in Supplementary Table S1 with previously described thermal profiles (Forghani et al., 2014; Gao et al., 2018). The PCR mixture (25 μL) contained of 50 ng of genomic DNA, 2 μM of each primer, and 12.5 μL of PCR Premix TaqTM (Takara, China). The amplification and agarose gel electrophoresis analysis were performed as described previously (Gao et al., 2018). DNA fingerprints were analyzed by Bionumerics software version 7.6 (Applied Maths, Belgium). The result of ERIC-PCR fingerprinting was used to characterize isolates from the same sample in order to exclude clonal isolates.

### Antimicrobial Susceptibility Testing

The sensitivity of *B. cereus* to 20 antimicrobial agents was tested using the standard Kirby-Bauer disk diffusion method (Park et al., 2009; The Clinical and Laboratory Standards Institute [CLSI], 2010; Kim et al., 2015; Gao et al., 2018; Osman et al., 2018; Yu et al., 2019). The *B. cereus* isolates were streaked on the nutrition agar plate and grown 16–18 h at 37°C. The colony was then picked and suspended using 0.85% physiological saline to 0.5 McFarland standard and spread on the surface of a Mueller-Hinton agar plate. After the inoculum was dried, the antimicrobial disks were put to the surface of the plates. The Mueller-Hinton agar plates were incubated 16–18 h at 35 ± 2°C, and the inhibition zone was measured. The isolates were classed as susceptible (S), intermediate (I), or resistant (R) according to CLSI guidelines and the inhibition zones were measured and interpreted according to the zone diameter interpretation criteria for *S. aureus* in Supplementary Table S2.

### Housekeeping Gene Amplification, Sequencing, and Sequence Type Determination

The genetic diversity of the *B. cereus* isolates was characterized using multilocus sequence typing (MLST) with primers specific for seven housekeeping genes (*glp, gmk, ilv, pta, pur, pyc*, and *tpi*; Supplementary Table S1). The seven housekeeping genes were sequenced by BGI (Shenzhen, China) and submitted to the PubMLST database for allele number identification. Each isolate was assigned a sequence type (ST) according to their combination of the seven housekeeping gene alleles. New STs were validated by the MLST database curator. Clonal complexes (CCs) were defined as single locus variants (SLV) of two or more independent isolates that shared identical alleles at six or seven loci (Drewnowska and Swiecicka, 2013) and were identified using the geoBURST tool (Francisco et al., 2009) with bootstrap resampling (1,000). Evolutionary relationships between the isolates were evaluated using a minimum spanning tree constructed by PHYLOViZ 2.0 software (Instituto de Microbiologia, Portugal; Ribeiro-Goncalves et al., 2016).

## Results

### Prevalence of *B. cereus* in RTE Foods

In this study, 302 of the 860 collected samples (35%) were positive for *B. cereus*, including 224 of the 656 cooked meat samples (34%), 59 of the 119 rice/noodle samples (50%), and 19 of the 85 cold vegetable dishes in sauce samples (22%). 368 different strains of *B. cereus* were identified based on the results of biochemical analysis and ERIC-PCR fingerprinting (Supplementary Figure S2). Based on quantitative analysis, 68% (206/302) of the positive samples were contaminated at levels ranging between 3 and 1100 MPN/g; however, 10% (29/302) of the samples exceeded 1100 MPN/g, including 18 cooked meat samples, 10 rice/noodles samples and one cold vegetable dish in sauce sample (Table 1).

**TABLE 1:** Prevalence and contamination level of *B cereus* in different ready-to-eat foods.

**Type**	**Contamination rate (%)^a^**	**MPN value (MPN/g)^b^**		
		
		**MPN < 3 (%)**	**3 ≤ MPN < 1100 (%)**	**1100 ≤ MPN (%)**
Cooked meat	224/656(34)	55/224(25)	151/224(67)	18/224(8)
Rice/noodles	59/119(50)	10/59(17)	39/59(66)	10/59(17)
Cold vegetable dishes in sauce	19/85(22)	2/19(11)	16/19(84)	1/19(5)
Total	302/860(35)	67/302(22)	206/302(68)	29/302(10)

*^*a*^Contamination rate = Number of positive samples/Total samples. ^*b*^MPN value (MPN/g) = Most probable number of *B. cereus* per gram sample.*

### Distribution of Virulence Genes Among *B. cereus* Isolates

**FIGURE 1:**
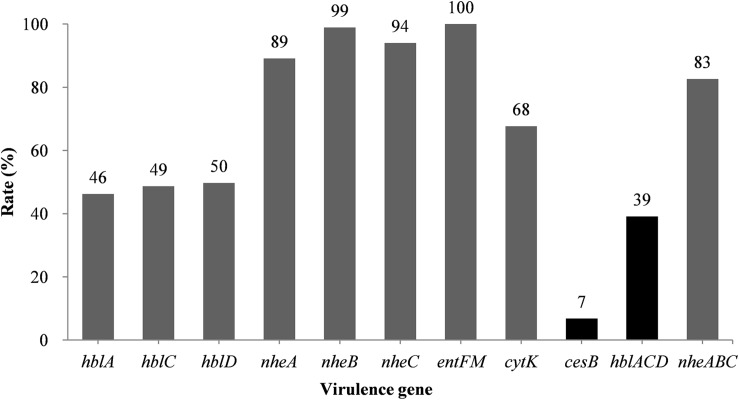
Distribution of virulence genes in *B. cereus* isolated from ready-to-eat foods in China. The number at the top of the bars represents the positivity rate for the corresponding toxin genes. *hblACD* and *nheABC* indicate strains positive for *hblA*, *hblC*, and *hblD*, or *nheA*, *nheB*, and *nheC*, respectively.

The *hblACD* gene cluster was found in 39% of the *B. cereus* isolates (Figure 1 and Supplementary Table S3), with *hblA*, *hblC*, and *hblD* present in 46, 49, and 50% of the isolates, respectively. The NHE genes *nheA*, *nheB*, and *nheC* were found in 89, 99, and 94% of the isolates, respectively. Moreover, 68% of the strains possessed the *cytK* gene, whereas the *cesB* gene was the least frequently observed toxin gene, present in just 7% of the strains. However, all of *B. cereus* isolates possessed the *entFM* gene, a higher rate than that of other enterotoxin genes.

**FIGURE 2:**
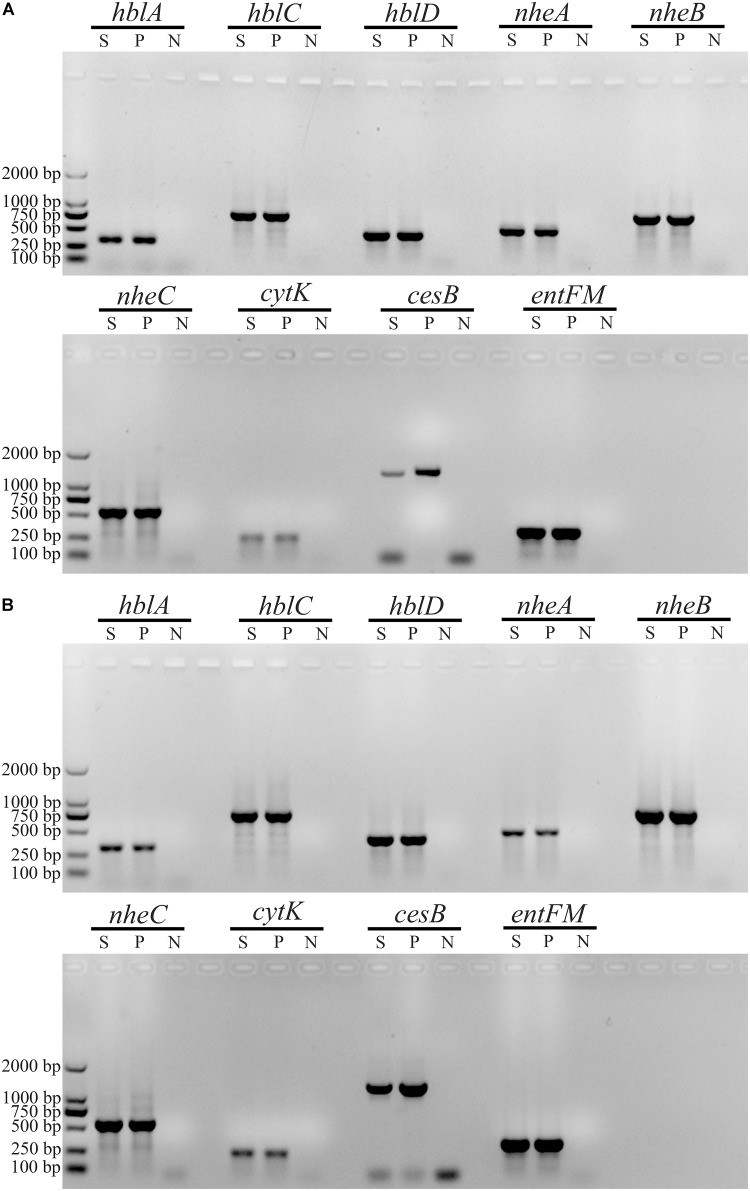
Characterization of virulence genes in *B. cereus* strains 2087-2-Bc and 3709-1A-Bc. Gel electrophoresis of PCR products of different virulence genes in *B. cereus* 2087-2-Bc **(A)** and *B. cereus* 3709-1A-Bc **(B)**. S, sample; P, positive control; N, negative control.

We observed 38 different virulence gene distribution spectra. The most abundant genetic profile, present in 33% of the strains, harbored eight virulence genes (*hblA*-*hblC*-*hblD*-*nheA*-*nheB*-*nheC*-*entFM*-*cytK*). Only two isolates (2087-2-Bc and 3709-1A-Bc, Figure 2) contained all nine virulence genes, whereas 27 isolates harbored just three virulence genes each (*nheA*-*nheB*-*entFM*, *nheA*-*entFM*-*cytK*, or *nheB*-*nheC*-*entFM*).

### Antimicrobial Susceptibility of *B. cereus* Isolates

**FIGURE 3:**
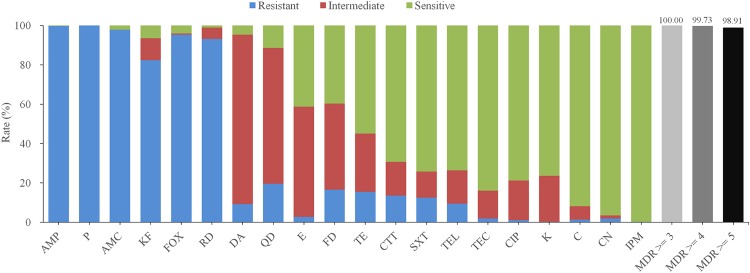
Antibiotic susceptibility of 368 *B. cereus* strains isolated from ready-to-eat foods in China. Different colors bars (blue, red, and green) represent the proportion of resistant, moderately resistant, and sensitive strains, respectively. The proportion of multidrug resistance (MDR) strains resistant to at least three, four, or five antibiotics is indicated by light gray, gray, or dark bars, respectively. AMP, ampicillin; P, penicillin; AMC, aoxicillin-clavulanic acid; KF, cephalothin; FOX, cefoxitin; RD, rifampin; DA, clindamycin; QD, quinupristin-dalfopristin; E, erythromycin; FD, nitrofurantoin; TE, tetracycline; CTT, cefotetan; SXT, trimethoprim-sulfamethoxazole; TEL, telithromycin; TEC, teicoplanin; CIP, ciprofloxacin; K, kanamycin; C, chloramphenicol; CN, gentamicin; IPM, imipenem.

The antimicrobial sensitivity results of the *B. cereus* isolates are shown in Figure 3 and Table 2. The isolates displayed different degrees of resistance to different antibiotics, particularly β-lactams. The highest rate of resistance was to penicillin (P; 100%), followed by ampicillin (AMP; 99.73%), amoxicillin-clavulanic acid (AMC; 97.83%), cefoxitin (FOX; 95.38%), and cephalothin (KF; 82.34%). Moreover, 13.59% of the strains were resistant to cefotetan (CTT). Most of the isolates were also resistant to rifampin (RD; 93.21%), but far fewer were resistant to quinupristin-dalfopristin (QD; 19.57%), nitrofurantoin (FD; 16.58%), tetracycline (TET; 15.49%) and trimethoprim-sulfamethoxazole (SXT; 12.50%). All isolates were sensitive to other antibiotics, including gentamicin (CN; 96.47%), teicoplanin (TEC; 83.97%), ciprofloxacin (CIP; 78.80%), kanamycin (K; 76.36%), and telithromycin (TEL; 73.64%).

**TABLE 2:** Antibiotic susceptibility of 368 *B. cereus* strains isolated from ready-to-eat foods in China.

**Category**	**Antimicrobial class**		**Antimicrobial agents**	***Bacillus cereus* (*n* = 368)**		
				
				**Resistant**	**Intermediate**	**Sensitive**
β-Lactams	Penicillins	I	Ampicillin (10 μg)	367(99.73%)	0(0.00%)	1(0.27%)
		II	Penicillin (10 units)	368(100.00%)	0(0.00%)	0(0.00%)
	β-Lactam/β-lactamase inhibitor combinations	III	Amoxicillin-clavulanic acid (20 μg/10 μg)	360(97.83%)	0(0.00%)	8(2.17%)
	Cephems (parenteral)	IV	Cephalothin (30 μg)	303(82.34%)	41(11.14%)	24(6.52%)
		V	Cefoxitin (30 μg)	351(95.38%)	2(0.54%)	15(4.08%)
		VI	Cefotetan (30 μg)	50(13.59%)	63(17.12%)	255(62.29%)
	Penems	VII	Imipenem (10 μg)	1(0.27%)	0(0.00%)	367(99.73%)
Non β-Lactams	Aminoglycosides	VIII	Gentamicin (10 μg)	7(1.90%)	6(1.63%)	355(96.47%)
		IX	Kanamycin (30 μg)	1(0.27%)	86(23.37%)	281(76.36%)
	Macrolides	X	Erythromycin (15 μg)	10(2.72%)	206(55.98%)	152(41.30%)
	Ketolide	XI	Telithromycin (15 μg)	35(9.51%)	62(16.85%)	271(73.64%)
	Glycopeptides	XII	Teicoplanin (30 μg)	7(1.90%)	52(14.13%)	309(83.97%)
	Quinolones	XIII	Ciprofloxacin (5 μg)	4(1.09%)	74(20.11%)	290(78.80%)
	Phenylpropanol	XIV	Chloramphenicol (30 μg)	5(1.36%)	25(6.79%)	338(91.85%)
	Tetracyclines	XV	Tetracycline (30 μg)	57(15.49%)	109(29.62%)	202(54.89%)
	Folate pathway inhibitors	XVI	Trimethoprim-Sulfamethoxazole (1.25 μg/23.75 μg)	46(12.50%)	49(13.32%)	273(74.18%)
	Lincosamides	XVII	Clindamycin (2 μg)	34(9.24%)	317(86.14%)	17(4.62%)
	Ansamycins	XVIII	Rifampin (5 μg)	343(93.21%)	21(5.71%)	4(1.09%)
	Streptogramins	XIX	Quinupristin-dalfopristin (15 μg)	72(19.57%)	254(69.02%)	42(11.41%)
	Nitrofurans	XX	Nitrofurantoin (300 μg)	61(16.58%)	161(43.75%)	146(39.67%)
Pansusceptible			≥3 Antimicrobia	100.00%		
			≥4 Antimicrobia	99.73%		
			≥5 Antimicrobia	98.91%		

Notably, two isolates were resistant to 12 of the antibiotics tested ampicillin (AMP), amoxicillin-clavulanic acid (AMC), penicillin (P), cephalothin (KF), cefoxitin (FOX), cefotetan (CTT), teicoplanin (TEC), trimethoprim-sulfamethoxazole (SXT), clindamycin (DA), rifampin (RD), quinupristin-dalfopristin (QD), nitrofurantoin (FD) and ampicillin (AMP), amoxicillin-clavulanic acid (AMC), penicillin (P), cephalothin (KF), cefoxitin (FOX), erythromycin (E), telithromycin (TEL), trimethoprim-sulfamethoxazole (SXT), clindamycin (DA), rifampin (RD), quinupristin-dalfopristin (QD), nitrofurantoin (FD), respectively. Whilst > 29.35% of the strains were resistant to the six most commonly used antibiotics (AMP-AMC-P-KF-FOX-RD). According to the definition of multidrug resistance (MDR) (Magiorakos et al., 2012), all isolated *B. cereus* strains were qualified as MDR strains and > 98.91% of the isolates were resistant to five or more antimicrobials.

### MLST and Clonal Complex Analysis

**FIGURE 4:**
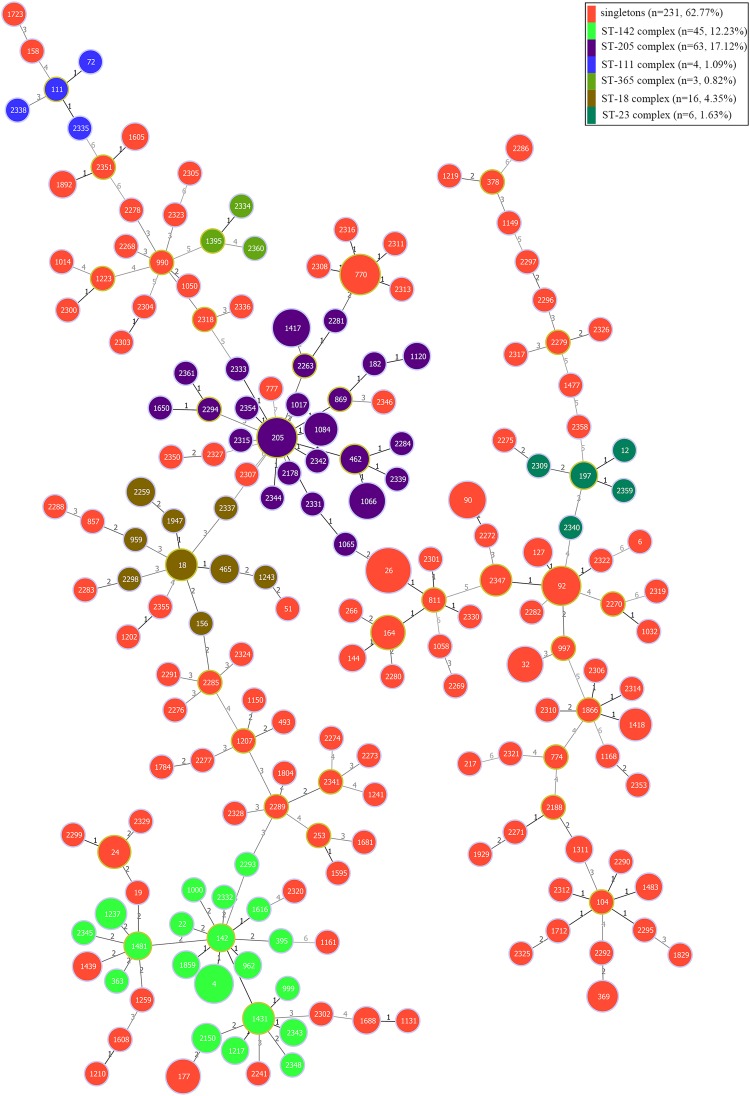
Minimum spanning tree and genetic diversity of 368 *B. cereus* strains isolated from ready-to-eat foods in China. Circles filled with red color represent different singletons and with other different colors represent different clonal complexes. The numbers inside the circle indicates the corresponding sequence type (ST). The color grading and corresponding number of the line indicate the change in seven sites between the two strains at both ends of the line. The circle with a larger diameter in each clonal complex is represented the dominant ST.

The genetic diversity of the 368 *B. cereus* isolates was analyzed by MLST with the internal fragment sequences of seven housekeeping genes. In total, 192 different STs were identified, 93 of which were novel and designated ST2150–ST2361. ST26 was the most abundant ST (28 isolates), followed by ST205 (14 isolates). Based on geoBURST analysis, the 192 different STs were grouped into six clonal complexes (CCs; ST-18, ST-23, ST-111, ST-142, ST-205, and ST-365) and 127 singletons. Among these CCs, ST-205, and ST-142 were predominant. Phylogenetic analysis was performed using the concatenated sequences of the seven housekeeping genes; in ST-205 complex, ST205 was the main evolutionary starter and 23 singletons evolved (Figure 4), whereas ST142 was the main evolutionary starter in ST-142 complex.

## Discussion

### Prevalence and Genetic Diversity of *B. cereus* Isolates

*Bacillus cereus* is a foodborne pathogen that causes various symptoms and is found on multiple types of food. In this study, 302 of the 860 RTE samples (35%) were positive for *B. cereus*, indicating that retailed RTE foods are a potential risk to consumers. The rate of contamination observed in this study was higher than has been reported in some countries, for example Korea (Cho et al., 2011) and Morocco (Merzougui et al., 2014), but lower than in others such as India (Sudershan et al., 2012). Around 90% of the samples had <1100 MPN/g of *B. cereus*, the generally accepted threshold for *B. cereus* contamination; however, the remaining samples all exceeded 1100 MPN/g, indicating that some RTE foods may cause food poisoning due to high levels of contamination.

Cooked meats are commonly prepared and sold for direct consumption in different nations. In this study, *B. cereus* was present in 34% (220/656) of the cooked meat samples, consistent with the frequency reported by Tewari et al. (2015). Research has shown that open-air stalls increases the opportunity for environmental pollution (Ng et al., 2013); for instance, cooked meat sold in open-air stalls can be exposed to dust-containing spores, increasing the chance of *B. cereus* contamination. This could explain the high prevalence of *B. cereus* in our cooked meat samples.

Vomiting was first associated with *B. cereus* in the United Kingdom in 1971 which was caused by fried rice (Mortimer and McCann, 1974). Vomiting due to *B. cereus* infection is typically associated with starch-containing foods (Fricker et al., 2007; Delbrassinne et al., 2015) which are believed to promote the growth of *B. cereus* and its production of emetic toxins (Griffiths and Schraft, 2017). In this study, 50% (59/119) of the rice/noodle samples tested positive for *B. cereus*, which is higher than that reported in other studies of starch-containing foods (Chang et al., 2011; Delbrassinne et al., 2012; Merzougui et al., 2014).

Cold vegetable dishes in sauce, also known as Chinese salad, are a favorite RTE food in China. *B. cereus* was detected in 22% (19/85) of the cold vegetable dishes in sauce samples, a higher prevalence than observed in other reports (Valero et al., 2007), but lower than that observed in vegetable salads in Korea (Chon et al., 2015). Although cold vegetable dishes in sauce RTEs undergo processing steps, they are usually not subjected to heat processing; thus, microorganisms cannot be completely eliminated on the fresh vegetables. Some studies have indicated that the growth, harvest, processing, and packaging of vegetables are likely to increase the possibility of microbial contamination (Sagoo et al., 2003). Since *B. cereus* is widespread in nature, particularly in soil, vegetables are easily contaminated by this bacterium; previously, we showed that *B. cereus* was present in up to 50% of vegetables (Yu et al., 2019). Taken together, these results suggest that the consumption of cold vegetable dishes in sauce made from vegetables contaminated with *B. cereus* could increase the potential risk to the public.

Some studies have indicated that RTE foods can become contaminated by different bacterial pathogens via food preparation surfaces (Christison et al., 2008; Cho et al., 2011; de Oliveira et al., 2011; Shiningeni et al., 2019). Many pathogenic bacteria such as *B. cereus* are capable of forming viscous, highly heat- and drought-resistant spores. These properties increase spore retention and make their removal from preparation surfaces on production lines difficult. Following its preparation, the load of *B. cereus* in an RTE can increase rapidly and reach dangerous levels (above 10^3^ CFU/g or mL); thus, consumers face a higher risk of foodborne illness from RTE foods, which are usually eaten without additional heat treatment. Consequently, regulations have been imposed on the number of *B. cereus* cells in RTE foods in different countries and regions based on standard guidelines. Hong Kong classifies RTE foods as either satisfactory (<10^3^ CFU/g), acceptable (10^3^–10^5^ CFU/g), or unsatisfactory (>10^5^ CFU/g), and the sale of “unsatisfactory” RTE foods must be halted immediately (Food and Environmental Hygiene Department, 2014). The Food Standards of Australia and New Zealand classes RTEs into four levels: satisfactory, acceptable, unsatisfactory, and potentially harmful (>10^4^ CFU/g of *B. cereus*) (New South Wales Food Authority, 2009), whereas in England “unsatisfactory” RTE foods are those with >10^5^ CFU/g of *B. cereus* (Health Protection Agency, 2009). In this study, 29 positive samples (29/302, 10%) had more than 1100 MPN/g of *B. cereus*, which may exceed some of these guideline limits.

### Virulence Gene Profiles of *B. cereus* Isolates

The most common enterotoxin-encoding genes in the *B. cereus* strains we identified were the *nhe* and *entFM*, consistent with previous reports (Kim et al., 2010; Chon et al., 2015; Hwang and Park, 2015; Glasset et al., 2016; Gao et al., 2018; Yu et al., 2019). CytK is a cytotoxin isolated from a *B. cereus* strain that caused a severe food poisoning outbreak leading to three deaths in France (Lund et al., 2000). The *cytK* gene was present in 68% of the *B. cereus* isolates identified in this study, consistent with earlier studies that found the gene in 40–73% of *B. cereus* strains isolated from foods (Hwang and Park, 2015; Lee et al., 2017; Gao et al., 2018). Hence, the widespread distribution of diarrhea-causing *B. cereus* in RTE foods and its potential hazards cannot be ignored.

Approximately 7% (25/368) of the strains isolated in this study were emetic strains, similar to the findings of a study performed in Jordan (Batchoun et al., 2011), lower than the levels reported in Holland and Ghana (Biesta-Peters et al., 2016; Owusu-Kwarteng et al., 2017), but higher than found by studies in Korea (Chon et al., 2015; Hwang and Park, 2015). According to previous studies, starch-containing foods promote toxin production by *B. cereus* (Griffiths and Schraft, 2017). Interestingly, in this study the 18 *B. cereus* strains isolated from cooked meat contained the emetic toxin gene *cesB*. Since vomiting intoxication cases have been known to cause death (Mahler et al., 1997; Dierick et al., 2005; Lopez et al., 2015), even this comparatively small number of *cesB* positive strains cannot be ignored.

### Antimicrobial Susceptibility of *B. cereus*

Beyond food poisoning, *B. cereus* is also associated with non-gastrointestinal infections. Antibiotic susceptibility testing can provide a reference for the clinical treatment of food poisoning. In this study, we exhaustively tested the antimicrobial resistance of all *B. cereus* isolates. More than 82.34% of the isolates were resistant to most of the β-lactam antibiotics, whilst only 13.59% (50/368) and 0.27% (1/368) were resistant to CTT and imipenem (IPM), respectively. This result is unsurprising, since *B. cereus* can produce a β-lactamase (Chen et al., 2003; Bottone, 2010). According to the results of our antimicrobial sensitivity testing, suspected *B. cereus* infections should not be treated clinically with broad-spectrum cephalosporins and penicillin, but with the CTT or IPM. We also found that many *B. cereus* isolates displayed multiple-drug resistance profiles, suggesting that *B. cereus* infections in RTE foods pose a significant potential risk.

### Genetic Diversity of *B. cereus* in RTE Foods

Epidemiological typing methods, such as MLST, are considered crucial for studying the prevalence of foodborne bacteria. MLST is a nucleotide sequence-based approach for the unambiguous characterization of isolates that has been broadly used for epidemiological typing and risk analyses of many pathogenic bacteria, such as *S*. *aureus* (Wu et al., 2018), *L. monocytogenes* (Wu et al., 2016), *Cronobacter* spp. (Xu et al., 2015), Salmonella (Matheson et al., 2010), and *B. cereus* (Cardazzo et al., 2008; Yang et al., 2017; Zhang et al., 2017; Yu et al., 2019). In this study, we used MLST to analyze the genetic polymorphisms of *B*. *cereus* strains isolated from RTE foods (Supplementary Table S4). Apart from six CCs, most isolates were assigned as singletons; ST-205 and ST-142 were the predominant CCs, consistent with previous reports (Zhang et al., 2017). Moreover, 28 strains were assigned to ST26, including 18 strains that were found to contain the *cesB* gene. ST26 is a recognized ST associated with food poisoning that causes vomiting, and includes two clinically isolated strains (NC7401 and F4810/72) (Agata et al., 2002; Priest et al., 2004; Fricker et al., 2007), suggesting that potentially harmful strains may be present in RTE foods in China; however, further studies investigating cereulide production by these strains must be carried out to understand their threat.

## Conclusion

In this study, we evaluated the prevalence of *B. cereus* in RTE foods from different regions in China. Our results indicated that RTE foods are highly contaminated with *B. cereus* and that the isolated strains contained a variety of pathogenic genes which may increase the potential risk of foodborne diseases. In addition, most of the strains exhibited MDR profiles with important implications for clinical treatment. Together with the diverse genotypic polymorphisms we observed, our findings reveal the potential high risk of *B. cereus* in RTE foods.

## Data Availability Statement

All datasets generated for this study are included in the article/Supplementary Material.

## Author Contributions

YD, QW, JW, JZ, and SY conceived the project and designed the experiments. SY, PY, JW, CL, HG, CCL, LK, LY, SW, TL, MC, HZ, RP, YZ, and XW performed the experiments. QW and YD supervised the project. SY and YD analyzed the data and wrote the manuscript. QW, JW, and YD complemented the writing.

## Conflict of Interest

The authors declare that the research was conducted in the absence of any commercial or financial relationships that could be construed as a potential conflict of interest.

## References

[B1] Agata N. Ohta M. Yokoyama K. (2002). Production of *Bacillus cereus* emetic toxin (cereulide) in various foods. *Int. J. Food Microbiol.* 73 23–27. 10.1016/S0168-1605(01)00692-4 11883672

[B2] Batchoun R. Al-Sha’er A. I. Khabour O. F. (2011). Molecular characterization of *Bacillus cereus* toxigenic strains isolated from different food matrices in Jordan. *Foodborne Pathog. Dis.* 8 1153–1158. 10.1089/fpd.2011.0853 21714637

[B3] Beecher D. J. Schoeni J. L. Wong A. C. (1995). Enterotoxic activity of hemolysin BL from *Bacillus cereus*. *Infect. Immun*. 63 4423–4428. 7591080 10.1128/iai.63.11.4423-4428.1995 PMC173629

[B4] Berthold-Pluta A. Pluta A. Garbowska M. Stefanska I. (2019). Prevalence and toxicity characterization of *Bacillus cereus* in food products from Poland. *Foods* 8:269. 10.3390/foods8070269 31331094 PMC6678163

[B5] Biesta-Peters E. G. Dissel S. Reij M. W. Zwietering M. H. in’t Veld P. H. (2016). Characterization and exposure assessment of emetic *Bacillus cereus* and cereulide production in food products on the Dutch market. *J. Food Prot.* 79 230–238. 10.4315/0362-028X.JFP-15-217 26818983

[B6] Bonerba E. Di Pinto A. Novello L. Montemurro F. Terio V. Colao V. (2010). Detection of potentially enterotoxigenic food-related *Bacillus cereus* by PCR analysis. *Int. J. Food Sci. Technol.* 45 1310–1315. 10.1111/j.1365-2621.2010.02257.x

[B7] Bottone E. J. (2010). *Bacillus cereus*, a volatile human pathogen. *Clin. Microbiol. Rev.* 23 382–398. 10.1128/CMR.00073-09 20375358 PMC2863360

[B8] Cardazzo B. Negrisolo E. Carraro L. Alberghini L. Patarnello T. Giaccone V. (2008). Multiple-locus sequence typing and analysis of toxin genes in *Bacillus cereus* food-borne isolates. *Appl. Environ. Microbiol.* 74 850–860. 10.1128/AEM.01495-07 18083872 PMC2227710

[B9] Chang H. J. Lee J. H. Han B. R. Kwak T. K. Kim J. (2011). Prevalence of the levels of *Bacillus cereus* in fried rice dishes and its exposure assessment from Chinese-style restaurants. *Food Sci. Biotechnol.* 20 1351–1359. 10.1007/s10068-011-0186-3

[B10] Chen Y. Succi J. Tenover F. C. Koehler T. M. (2003). β-Lactamase genes of the penicillin-susceptible *Bacillus anthracis* sterne strain. *J. Bacteriol.* 185 823–830. 10.1128/JB.185.3.823-830.2003 12533457 PMC142833

[B11] Cho J. I. Lee S. H. Lim J. S. Koh Y. J. Kwak H. S. Hwang I. G. (2011). Detection and distribution of food-borne bacteria in ready-to-eat foods in Korea. *Food Sci. Biotechnol.* 20 525–529. 10.1007/s10068-011-0073-y

[B12] Chon J. W. Yim J. H. Kim H. S. Kim D. H. Kim H. Oh D. H. (2015). Quantitative prevalence and toxin gene profile of *Bacillus cereus* from ready-to-eat vegetables in South Korea. *Foodborne Pathog. Dis.* 12 795–799. 10.1089/fpd.2015.1977 26317539

[B13] Christison C. A. Lindsay D. von Holy A. (2008). Microbiological survey of ready-to-eat foods and associated preparation surfaces in retail delicatessens, Johannesburg, South Africa. *Food Control* 19 727–733. 10.1016/j.foodcont.2007.07.004

[B14] de Oliveira M. A. de Souza V. M. Bergamini A. M. M. De Martinis E. C. P. (2011). Microbiological quality of ready-to-eat minimally processed vegetables consumed in Brazil. *Food Control* 22 1400–1403. 10.1016/j.foodcont.2011.02.020

[B15] Delbrassinne L. Andjelkovic M. Dierick K. Denayer S. Mahillon J. Van Loco J. (2012). Prevalence and levels of *Bacillus cereus* emetic toxin in rice dishes randomly collected from restaurants and comparison with the levels measured in a recent foodborne outbreak. *Foodborne Pathog. Dis.* 9 809–814. 10.1089/fpd.2012.1168 22891880

[B16] Delbrassinne L. Botteldoorn N. Andjelkovic M. Dierick K. Denayer S. (2015). An emetic *Bacillus cereus* outbreak in a kindergarten: detection and quantification of critical levels of cereulide toxin. *Foodborne Pathog. Dis.* 12 84–87. 10.1089/fpd.2014.1788 25457101

[B17] Dierick K. Van Coillie E. Swiecicka I. Meyfroidt G. Devlieger H. Meulemans A. (2005). Fatal family outbreak of *Bacillus cereus*-associated food poisoning. *J. Clin. Microbiol.* 43 4277–4279. 10.1128/JCM.43.8.4277-4279.2005 16082000 PMC1233987

[B18] Doménech-Sánchez A. Laso E. Pérez M. J. Berrocal C. I. (2011). Emetic disease caused by *Bacillus cereus* after consumption of tuna fish in a beach club. *Foodborne Pathog. Dis.* 8 835–837. 10.1089/fpd.2010.0783 21381943

[B19] Drewnowska J. M. Swiecicka I. (2013). Eco-genetic structure of *Bacillus cereus* sensu lato populations from different environments in northeastern Poland. *PLoS One* 8:e80175. 10.1371/journal.pone.0080175 24312460 PMC3846478

[B20] Ehling-Schulz M. Frenzel E. Gohar M. (2015). Food-bacteria interplay: pathometabolism of emetic *Bacillus cereus*. *Front. Microbiol.* 6:704. 10.3389/fmicb.2015.00704 26236290 PMC4500953

[B21] Ehling-Schulz M. Vukov N. Schulz A. Shaheen R. Andersson M. Martlbauer E. (2005). Identification and partial characterization of the nonribosomal peptide synthetase gene responsible for cereulide production in emetic *Bacillus cereus*. *Appl. Environ. Microbiol.* 71 105–113. 10.1128/AEM.71.1.105-113.2005 15640177 PMC544239

[B22] Food and Environmental Hygiene Department (2014). *Microbiological Guidelines for Food.* Hong Kong: Food and Environmental Hygiene Department.

[B23] Forghani F. Kim J. B. Oh D. H. (2014). Enterotoxigenic profiling of emetic toxin-and enterotoxin-producing *Bacillus cereus*, isolated from food, environmental, and clinical samples by multiplex PCR. *J. Food Sci.* 79 M2228–M2293. 10.1111/1750-3841.12666 25311736

[B24] Francisco A. P. Bugalho M. Ramirez M. Carriço J. A. (2009). Global optimal eBURST analysis of multilocus typing data using a graphic matroid approach. *BMC Bioinformatics* 10:152. 10.1186/1471-2105-10-152 19450271 PMC2705362

[B25] Fricker M. Messelhäusser U. Busch U. Scherer S. Ehling-Schulz M. (2007). Diagnostic real-time PCR assays for the detection of emetic *Bacillus cereus* strains in foods and recent food-borne outbreaks. *Appl. Environ. Microbiol.* 73 1892–1898. 10.1128/AEM.02219-06 17259359 PMC1828801

[B26] Friedman N. D. Temkin E. Carmeli Y. (2016). The negative impact of antibiotic resistance. *Clin. Microbiol. Infect.* 22 416–422. 10.1016/j.cmi.2015.12.002 26706614

[B27] Gao T. Ding Y. Wu Q. Wang J. Zhang J. Yu S. (2018). Prevalence, virulence genes, antimicrobial susceptibility, and genetic diversity of *Bacillus cereus* isolated from pasteurized milk in China. *Front. Microbiol.* 9:533. 10.3389/fmicb.2018.00533 29632521 PMC5879084

[B28] Glasset B. Herbin S. Guillier L. Cadel-Six S. Vignaud M. L. Grout J. (2016). *Bacillus cereus*-induced food-borne outbreaks in France, 2007 to 2014: epidemiology and genetic characterisation. *Euro Surveill.* 21:30413. 10.2807/1560-7917.ES.2016.21.48.30413 27934583 PMC5388111

[B29] Granum P. E. Lund T. (1997). *Bacillus cereus* and its food poisoning toxins. *FEMS Microbiol. Lett.* 157 223–228. 10.1016/S0378-1097(97)00438-2 9435100

[B30] Granum P. E. O’sullivan K. Lund T. (1999). The sequence of the non-haemolytic enterotoxin operon from *Bacillus cereus*. *FEMS Microbiol. Lett.* 177 225–229. 10.1111/j.1574-6968.1999.tb13736.x 10474188

[B31] Griffiths M. W. Schraft H. (2017). “‘’*Bacillus cereus* food poisoning”,” in *Foodborne Diseases*, eds Dodd C. E. R. Aldsworth T. Stein R. A. Cliver D. O. (London: Academic Press), 395–405. 10.1016/b978-0-12-385007-2.00020-6

[B32] Hansen B. M. Hendriksen N. B. (2001). Detection of enterotoxic *Bacillus cereus* and *Bacillus thuringiensis* strains by PCR analysis. *Appl. Environ. Microbiol.* 67 185–189. 10.1128/AEM.67.1.185-189.2001 11133444 PMC92543

[B33] Health Protection Agency (2009). *Guidelines for Assessing the Microbiological Safety of Ready-to-Eat Foods Placed on the Market [S/OL].* London: Health Protection Agency.

[B34] Hwang J. Y. Park J. H. (2015). Characteristics of enterotoxin distribution, hemolysis, lecithinase, and starch hydrolysis of *Bacillus cereus* isolated from infant formulas and ready-to-eat foods. *J. Dairy Sci.* 98 1652–1660. 10.3168/jds.2014-9042 25597976

[B35] Ikeda M. Yagihara Y. Tatsuno K. Okazaki M. Okugawa S. Moriya K. (2015). Clinical characteristics and antimicrobial susceptibility of *Bacillus cereus* blood stream infections. *Ann. Clin. Microbiol. Antimicrob.* 14:43. 10.1186/s12941-015-0104-2 26370137 PMC4570458

[B36] Jensen G. B. Hansen B. M. Eilenberg J. Mahillon J. (2003). The hidden lifestyles of *Bacillus cereus* and relatives. *Environ. Microbiol.* 5 631–640. 10.1046/j.1462-2920.2003.00461.x 12871230

[B37] Kim C. W. Cho S. H. Kang S. H. Park Y. B. Yoon M. H. Lee J. B. (2015). Prevalence, genetic diversity, and antibiotic resistance of *Bacillus cereus* isolated from Korean fermented soybean products. *J. Food Sci.* 80 M123–M128. 10.1111/1750-3841.12720 25472031

[B38] Kim J. B. Kim J. M. Kim C. H. Seo K. S. Park Y. B. Choi N. J. (2010). Emetic toxin producing *Bacillus cereus* Korean isolates contain genes encoding diarrheal-related enterotoxins. *Int. J. Food Microbiol.* 144 182–186. 10.1016/j.ijfoodmicro.2010.08.021 20869784

[B39] Lee N. Kim M. D. Chang H. J. Choi S. W. Chun H. S. (2017). Genetic diversity, antimicrobial resistance, toxin gene profiles, and toxin production ability of *Bacillus cereus* isolates from doenjang, a Korean fermented soybean paste. *J. Food Saf.* 37 e12363 10.1111/jfs.12363

[B40] Lopez A. C. Minnaard J. Perez P. F. Alippi A. M. (2015). A case of intoxication due to a highly cytotoxic *Bacillus cereus* strain isolated from cooked chicken. *Food Microbiol.* 46 195–199. 10.1016/j.fm.2014.08.005 25475284

[B41] Lund T. De Buyser M. L. Granum P. E. (2000). A new cytotoxin from *Bacillus cereus* that may cause necrotic enteritis. *Mol. Microbiol.* 38 254–261. 10.1046/j.1365-2958.2000.02147.x 11069652

[B42] Lund T. Granum P. E. (1996). Characterisation of a non-haemolytic enterotoxin complex from *Bacillus cereus* isolated after a foodborne outbreak. *FEMS Microbiol. Lett.* 141 151–156. 10.1111/j.1574-6968.1996.tb08377.x 8768516

[B43] Magiorakos A. P. Srinivasan A. Carey R. B. Carmeli Y. Falagas M. E. Giske C. G. (2012). Multidrug-resistant, extensively drug-resistant and pandrug-resistant bacteria: an international expert proposal for interim standard definitions for acquired resistance. *Clin. Microbiol. Infect.* 18 268–281. 10.1111/j.1469-0691.2011.03570.x 21793988

[B44] Mahler H. Pasi A. Kramer J. M. Schulte P. Scoging A. C. Bar W. (1997). Fulminant liver failure in association with the emetic toxin of *Bacillus cereus*. *N. Engl. J. Med.* 336 1142–1148. 10.1056/NEJM199704173361604 9099658

[B45] Marrollo R. (2016). “*Bacillus cereus* food-borne disease,” in *The Diverse Faces of Bacillus cereus*, ed. Savini V. (London: Academic Press), 61–72. 10.1016/b978-0-12-801474-5.00005-0

[B46] Matheson N. Kingsley R. A. Sturgess K. Aliyu S. H. Wain J. Dougan G. (2010). Ten years experience of *Salmonella* infections in Cambridge. *UK. J. Infect.* 60 21–25. 10.1016/j.jinf.2009.09.016 19819256

[B47] Merzougui S. Lkhider M. Grosset N. Gautier M. Cohen N. (2014). Prevalence, PFGE typing, and antibiotic resistance of *Bacillus cereus* group isolated from food in Morocco. *Foodborne Pathog. Dis.* 11 145–149. 10.1089/fpd.2013.1615 24206436

[B48] Mortimer P. R. McCann G. (1974). Food-poisoning episodes associated with *Bacillus cereus* in fried rice. *Lancet* 1 1043–1045. 10.1016/s0140-6736(74)90434-6 4133716

[B49] New South Wales Food Authority (2009). *Microbiological Quality Guide for Ready-to-Eat Foods. A Guide to Interpreting Microbiological Results [S/OL]. [2017-01-20]. NSW/FA/CP028/0906.* Newington, CT: New South Wales Food Authority.

[B50] Ng Y. F. Wong S. L. Cheng H. L. Yu H. F. Chan S. W. (2013). The microbiological quality of ready-to-eat food in Siu Mei and Lo Mei shops in Hong Kong. *Food Control* 34 547–553. 10.1016/j.foodcont.2013.05.018

[B51] Nicholls M. Purcell B. Willis C. Amar C. F. Kanagarajah S. Chamberlain D. (2016). Investigation of an outbreak of vomiting in nurseries in south east England. May 2012. *Epidemiol. Infect.* 144 582–590. 10.1017/S0950268815001491 26165194

[B52] Oltuszak-Walczak E. Walczak P. (2013). PCR detection of cytK gene in *Bacillus cereus* group strains isolated from food samples. *J. Microbiol. Methods* 95 295–301. 10.1016/j.mimet.2013.09.012 24060693

[B53] Osman K. M. Kappell A. D. Orabi A. Al-Maary K. S. Mubarak A. S. Dawoud T. M. (2018). Poultry and beef meat as potential seedbeds for antimicrobial resistant enterotoxigenic *Bacillus* species: a materializing epidemiological and potential severe health hazard. *Sci. Rep.* 8:11600. 10.1038/s41598-018-29932-3 30072706 PMC6072766

[B54] Owusu-Kwarteng J. Wuni A. Akabanda F. Tano-Debrah K. Jespersen L. (2017). Prevalence, virulence factor genes and antibiotic resistance of *Bacillus cereus* sensu lato isolated from dairy farms and traditional dairy products. *BMC Microbiol.* 17:65. 10.1186/s12866-017-0975-9 28288581 PMC5348786

[B55] Park Y. B. Kim J. B. Shin S. W. Kim J. C. Cho S. H. Lee B. K. (2009). Prevalence, genetic diversity, and antibiotic susceptibility of *Bacillus cereus* strains isolated from rice and cereals collected in Korea. *J. Food Prot.* 72 612–617. 10.4315/0362-028x-72.3.612 19343952

[B56] Priest F. G. Barker M. Baillie L. W. Holmes E. C. Maiden M. C. (2004). Population structure and evolution of the *Bacillus cereus* group. *J. Bacteriol.* 186 7959–7970. 10.1128/JB.186.23.7959-7970.2004 15547268 PMC529064

[B57] Rahimi E. Abdos F. Momtaz H. Baghbadorani Z. T. Jalali M. (2013). *Bacillus cereus* in infant foods: prevalence study and distribution of enterotoxigenic virulence factors in Isfahan province. Iran. *Sci. World J.* 2013:292571. 10.1155/2013/292571 23781153 PMC3678457

[B58] Ribeiro-Goncalves B. Francisco A. P. Vaz C. Ramirez M. Carriço J. A. (2016). PHYLOViZ Online: web-based tool for visualization, phylogenetic inference, analysis and sharing of minimum spanning trees. *Nucleic Acids Res.* 44 W246–W251. 10.1093/nar/gkw359 27131357 PMC4987911

[B59] Rishi E. Rishi P. Sengupta S. Jambulingam M. Madhavan H. N. Gopal L. (2013). Acute postoperative *Bacillus cereus* endophthalmitis mimicking toxic anterior segment syndrome. *Ophthalmology* 120 181–185. 10.1016/j.ophtha.2012.07.009 22986113

[B60] Sagoo S. K. Little C. L. Ward L. Gillespie I. A. Mitchell R. T. (2003). Microbiological study of ready-to-eat salad vegetables from retail establishments uncovers a national outbreak of salmonellosis. *J. Food Prot.* 66 403–409. 10.4315/0362-028x-66.3.403 12636292

[B61] Schmid D. Rademacher C. Kanitz E. E. Frenzel E. Simons E. Allerberger F. (2016). Elucidation of enterotoxigenic *Bacillus cereus* outbreaks in Austria by complementary epidemiological and microbiological investigations, 2013. *Int. J. Food Microbiol.* 232 80–86. 10.1016/j.ijfoodmicro.2016.05.011 27257745

[B62] Shiningeni D. Chimwamurombe P. Shilangale R. Misihairabgwi J. (2019). Prevalence of pathogenic bacteria in street vended ready-to-eat meats in Windhoek. *Namibia. Meat Sci.* 148 223–228. 10.1016/j.meatsci.2018.05.014 29861289

[B63] Sloan-Gardner T. S. Glynn-Robinson A. J. Roberts-Witteveen A. Krsteski R. Rogers K. Kaye A. (2014). An outbreak of gastroenteritis linked to a buffet lunch served at a Canberra restaurant. *Commun. Dis. Intell. Q. Rep.* 38 E273–E278. 25631587 10.33321/cdi.2014.38.45

[B64] Song Z. Zhao Q. Zhu L. Zhang Z. Jiang L. Huang H. (2019). Draft genome sequence of multidrug-resistant β-lactamase-producing *Bacillus cereus* S66 isolated from China. *J. Glob. Antimicrob. Resist.* 17 23–24. 10.1016/j.jgar.2019.02.019 30844497

[B65] Stenfors Arnesen L. P. Fagerlund A. Granum P. E. (2008). From soil to gut: *Bacillus cereus* and its food poisoning toxins. *FEMS Microbiol. Rev.* 32 579–606. 10.1111/j.1574-6976.2008.00112.x 18422617

[B66] Sudershan R. V. Naveen Kumar R. Kashinath L. Bhaskar V. Polasa K. (2012). Microbiological hazard identification and exposure assessment of poultry products sold in various localities of Hyderabad. India. *Sci World J.* 2012:736040. 10.1100/2012/736040 22593705 PMC3347783

[B67] Tallent S. M. Rhodehamel E. J. Harmon S. M. Bennett R. W. (2012). *Data from: Bacteriological analytical manual; methods for specific pathogens. U. S. Food and Drug Administration. Chapter 14 Bacillus cereus.* Available at: https://www.fda.gov/food/laboratory-methods-food/bam-bacillus-cereus (accessed November 7, 2019).

[B68] Tewari A. Singh S. P. Singh R. (2015). Incidence and enterotoxigenic profile of *Bacillus cereus* in meat and meat products of Uttarakhand. *India. J. Food Sci. Technol.* 52 1796–1801. 10.1007/s13197-013-1162-0 25745259 PMC4348265

[B69] The Clinical and Laboratory Standards Institute [CLSI] (2010). *Performance Standards for Antimicrobial Susceptibility Testing; Twentieth Informational Supplement. Approved Standard-M100-S20.* Wayne, PA: The Clinical and Laboratory Standards Institute.

[B70] The Hygiene Ministry of China (2003). *National Food Safety Standard. Food Microbiological Examination: Bacillus cereus Test.* Beijing: The Hygiene Ministry of China.

[B71] The Hygiene Ministry of China (2010). *National Food Safety Standard. Food Microbiological Examination: General Guidelines.* Beijing: The Hygiene Ministry of China.

[B72] Tran S. L. Guillemet E. Gohar M. Lereclus D. Ramarao N. (2010). CwpFM (EntFM) is a *Bacillus cereus* potential cell wall peptidase implicated in adhesion, biofilm formation, and virulence. *J. Bacteriol.* 192 2638–2642. 10.1128/JB.01315-09 20233921 PMC2863573

[B73] Valero M. Hernández-Herrero L. A. Giner M. J. (2007). Survival, isolation and characterization of a psychrotrophic *Bacillus cereus* strain from a mayonnaise-based ready-to-eat vegetable salad. *Food Microbiol.* 24 671–677. 10.1016/j.fm.2007.04.005 17613363

[B74] Wu S. Huang J. Wu Q. Zhang J. Zhang F. Yang X. (2018). *Staphylococcus aureus* isolated from retail meat and meat products in China: incidence, antibiotic resistance and genetic diversity. *Front. Microbiol.* 9:2767. 10.3389/fmicb.2018.02767 30498486 PMC6249422

[B75] Wu S. Wu Q. Zhang J. Chen M. Guo W. (2016). Analysis of multilocus sequence typing and virulence characterization of *Listeria monocytogenes* isolates from Chinese retail ready-to-eat food. *Front. Microbiol.* 7:168. 10.3389/fmicb.2016.00168 26909076 PMC4754575

[B76] Xu X. Li C. Wu Q. Zhang J. Huang J. Yang G. (2015). Prevalence, molecular characterization, and antibiotic susceptibility of *Cronobacter* spp. in Chinese ready-to-eat foods. *Int. J. Food Microbiol.* 204 17–23. 10.1016/j.ijfoodmicro.2015.03.003 25828706

[B77] Yang X. Zhang J. Yu S. Wu Q. Guo W. Huang J. (2016). Prevalence of *Staphylococcus aureus* and methicillin-resistant *Staphylococcus aureus* in retail ready-to-eat foods in China. *Front. Microbiol.* 7:816 10.3389/fmicb.2016.00816 PMC4895929 27375562

[B78] Yang Y. Yu X. Zhan L. Chen J. Zhang Y. Zhang J. (2017). Multilocus sequence type profiles of *Bacillus cereus* isolates from infant formula in China. *Food Microbiol.* 62 46–50. 10.1016/j.fm.2016.09.007 27889164

[B79] Yu P. Yu S. Wang J. Guo H. Zhang Y. Liao X. (2019). *Bacillus cereus* isolated from vegetables in China: incidence, genetic diversity, virulence genes, and antimicrobial resistance. *Front. Microbiol.* 10:948. 10.3389/fmicb.2019.00948 31156567 PMC6530634

[B80] Zhang Y. Chen J. Feng C. Zhan L. Zhang J. Li Y. (2017). Quantitative prevalence, phenotypic and genotypic characteristics of *Bacillus cereus* isolated from retail infant foods in China. *Foodborne Pathog. Dis.* 14 564–572. 10.1089/fpd.2017.2287 28753035

