# Supplementary figures and images for: A Study on Prevalence and Characterization of Bacillus cereus in Ready-to-Eat Foods in China
Source: Front Microbiol. 2020 Jan 15;10:3043. doi: 10.3389/fmicb.2019.03043 (PMC6974471; doi:10.3389/fmicb.2019.03043)

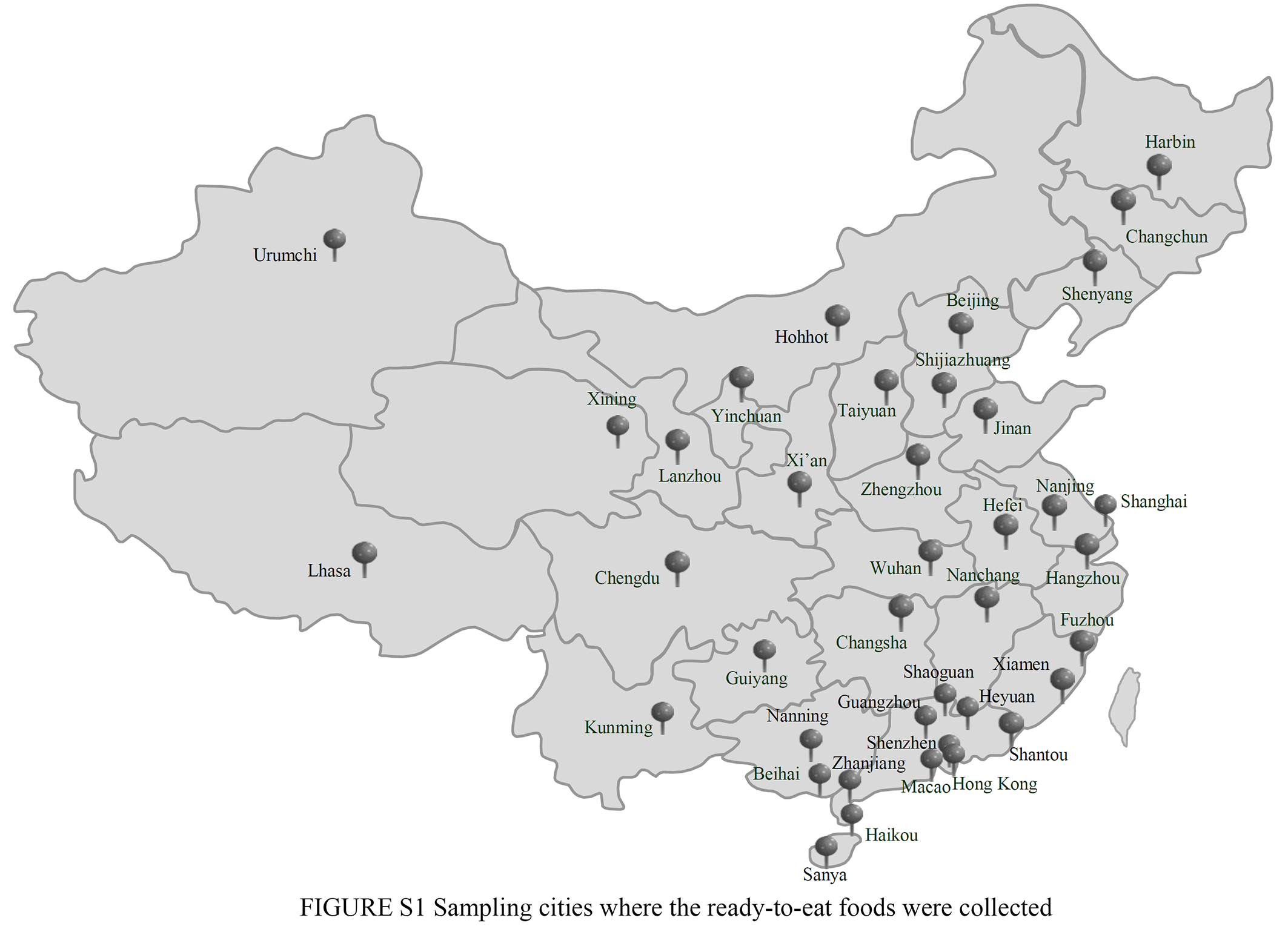

Supplement: Supplementary file 1 [file Image_1.TIF]

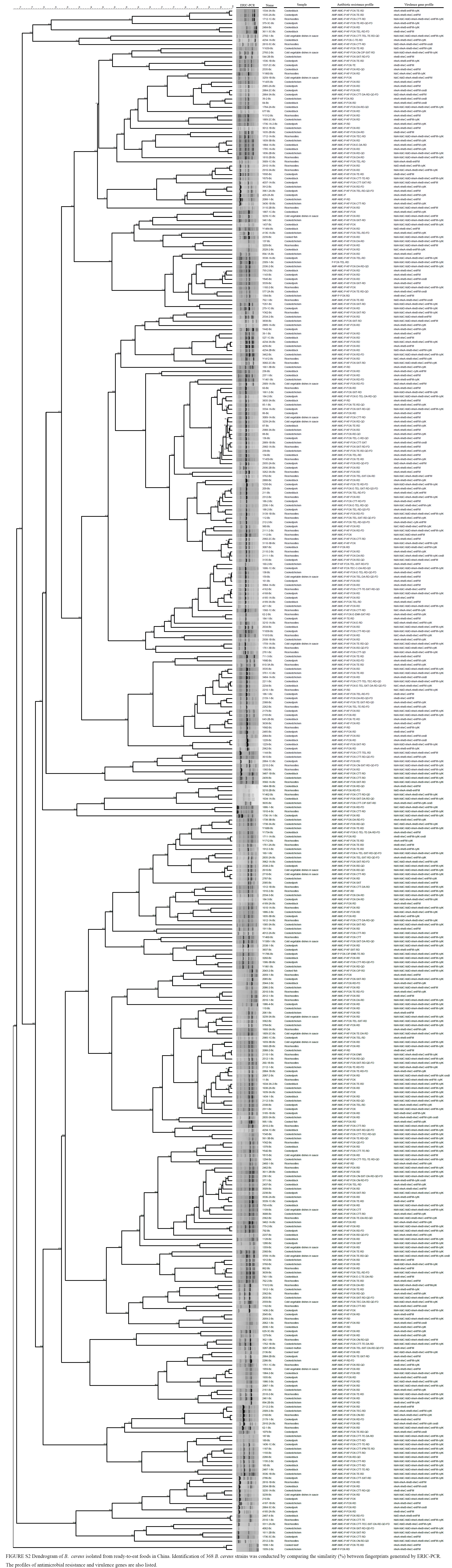

Supplement: Supplementary file 2 [file Image_2.tif]
