# Supplementary material for: A Study on Prevalence and Characterization of Bacillus cereus in Ready-to-Eat Foods in China
Source: Front Microbiol. 2020 Jan 15;10:3043. doi: 10.3389/fmicb.2019.03043 (PMC6974471; doi:10.3389/fmicb.2019.03043)
Supplement: Supplementary file 3 [file Table_1.DOCX]

**Supplementary Table 1** Primer list in this study

| **Primer** | **Sequence (5’-3’)** | **Target fragment length (bp)** | **Annealing temperature (℃)** | **Reference** |
| --- | --- | --- | --- | --- |
| HblA-F | GTGCAGATGTTGATGCCGAT | 320 | 55 | ([Hansen and Hendriksen, 2001](#_ENREF_25)) |
| HblA-R | ATGCCACTGCGTGGACATAT |  |  |  |
| HblC-F | AATGGTCATCGGAACTCTAT | 750 | 55 |  |
| HblC-R | CTCGCTGTTCTGCTGTTAAT |  |  |  |
| HblD-F | AATCAAGAGCTGTCACGAAT | 430 | 55 |  |
| HblD-R | CACCAATTGACCATGCTAAT |  |  |  |
| NheA-F | TACGCTAAGGAGGGGCA | 500 | 55 |  |
| NheA-R | GTTTTTATTGCTTCATCGGCT |  |  |  |
| NheB-F | CTATCAGCACTTATGGCAG | 770 | 55 |  |
| NheB-R | ACTCCTAGCGGTGTTCC |  |  |  |
| NheC-F | CGGTAGTGATTGCTGGG | 583 | 55 |  |
| NheC-R | CAGCATTCGTACTTGCCAA |  |  |  |
| entFM-F | CCACTGCAGTCAAAACCAGC | 327 | 58 | (Forghani et al., 2014) |
| entFM-R | AGGCCCAGCTACATACAACG |  |  |  |
| cytK-F | AAAATGTTTAGCATTATCCGCTGT | 238 | 55 | ([Oltuszak-Walczak and Walczak, 2013](#_ENREF_47)) |
| cytK-R | ACCAGTTGTATTAATAACGGCAATC |  |  |  |
| cesB-F | GGTGACACATTATCATATAAGGTG | 1271 | 58 | ([Ehling-Schulz et al., 2005](#_ENREF_17)) |
| cesB-R | GTAAGCGAACCTGTCTGTAACAACA |  |  |  |
| ERIC -F | ATGTAAGCTCCTGGGGATTCAC | 200 up | 45 | (Versalovic et al., 1991) |
| ERIC -R | AAGTAAGTGACTGGGGTGAGCG |  |  |  |
| glpF-F | GCGTTTGTGCTGGTGTAAGT | 549 | 59 | PubMLST (http://pubmlst.org/bcereus/info/primers.shtml) |
| glpF-R | CTG CAATCGGAAGGAAGAAG |  |  |  |
| gmk-F | TTAAGTGAGGAAGGGTAGG | 600 | 56 |  |
| gmk-R | AATGTTCACCAACCACAA |  |  |  |
| ilvD-F | GGGCAAACATTAAGAGAA | 556 | 58 |  |
| ilvD-R | TTCTGGTCGTTTCCATTC |  |  |  |
| pta-F | AGAGCGTTTAGCAAAAGAA | 576 | 56 |  |
| pta-R | CAATGCGAGTTGCTTCTA |  |  |  |
| pur-F | GCTGCGAAAAATCACAAA | 536 | 56 |  |
| pur-R | CACGATTCGCTGCAATAA |  |  |  |
| pycA-F | GTTAGGTGGAAACGAAAG | 550 | 57 |  |
| pycA-R | CGTCCAAGTTTATGGAAT |  |  |  |
| tpi-F | CCAGTAGCACTTAGCGAC | 553 | 58 |  |
| tpi-R | GAAACCGTCAAGAATGAT |  |  |  |
