# Supplementary material for: A Study on Prevalence and Characterization of Bacillus cereus in Ready-to-Eat Foods in China
Source: Front Microbiol. 2020 Jan 15;10:3043. doi: 10.3389/fmicb.2019.03043 (PMC6974471; doi:10.3389/fmicb.2019.03043)
Supplement: Supplementary file 5 [file Table_3.DOCX]

**Supplementary Table 3** Prevalence of virulence genes in *Bacillus cereus* isolated

from ready-to-eat foods in China

| Toxigenic genes | Number of strains (%) positive for target gene |
| --- | --- |
| Hemolysin BL genes |  |
| *hblA* | 170 (46%) |
| *hblC* | 179 (49%) |
| *hblD* | 183 (50%) |
| *hblACD* | 144 (39%) |
| Non-hemolytic enterotoxin genes |  |
| *nheA* | 328 (89%) |
| *nheB* | 364 (99%) |
| *nheC* | 346 (94%) |
| *nheABC* | 304 (83%) |
| Enterotoxin FM gene |  |
| *entFM* | 368 (100%) |
| Cytotoxin K gene |  |
| *cytK* | 249 (68%) |
| Cereulide synthetase gene |  |
| *cesB* | 25 (7%) |
| All nine genes | 2 (0.5%) |
