# Supplementary material for: A Study on Prevalence and Characterization of Bacillus cereus in Ready-to-Eat Foods in China
Source: Front Microbiol. 2020 Jan 15;10:3043. doi: 10.3389/fmicb.2019.03043 (PMC6974471; doi:10.3389/fmicb.2019.03043)
Supplement: Supplementary file 6 [file Table_4.DOCX]

**Supplementary Table 4** Information about genetic diversity of *B. cereus* isolated from ready-to-eat foods in China

| No. | Strain name | ST type | Clonal complex | No. | Strain name | ST type | Clonal complex | |
| --- | --- | --- | --- | --- | --- | --- | --- | --- |
| 1 | 11-Bc | 4 | ST-142 complex | 17 | 1762^-1^B-Bc | 18 | ST-18 complex | |
| 2 | 1634^-3^A-2-Bc | 4 | ST-142 complex | 18 | 2211-Bc | 18 | ST-18 complex | |
| 3 | 1638^-2^A-Bc | 4 | ST-142 complex | 19 | 1434-1-Bc | 19 | Singleton | |
| 4 | 1639^-3^A-Bc | 4 | ST-142 complex | 20 | 2510-2-Bc | 22 | ST-142 complex | |
| 5 | 1659^-3^B-Bc | 4 | ST-142 complex | 21 | 184-3-Bc | 24 | Singleton | |
| 6 | 1660^-2^B-Bc | 4 | ST-142 complex | 22 | 1280-Bc | 24 | Singleton | |
| 7 | 1736^-1^A-1-Bc | 4 | ST-142 complex | 23 | Y1688-Bc | 24 | Singleton | |
| 8 | 1738^-3^A-Bc | 4 | ST-142 complex | 24 | 2312-Bc | 24 | Singleton | |
| 9 | 1986-4-Bc | 4 | ST-142 complex | 25 | 4168-Bc | 24 | Singleton | |
| 10 | 2311-Bc | 4 | ST-142 complex | 26 | 39-2-Bc^#^ | 26 | Singleton | |
| 11 | 2336-2-Bc | 4 | ST-142 complex | 27 | 84-Bc^#^ | 26 | Singleton | |
| 12 | 2635-Bc | 4 | ST-142 complex | 28 | 577-2A-Bc^#^ | 26 | Singleton | |
| 13 | 677-Bc | 6 | Singleton | 29 | 762-1-Bc^#^ | 26 | Singleton | |
| 14 | 4037-1A-Bc | 12 | ST-23 complex | 30 | 793-2-Bc | 26 | Singleton | |
| 15 | Y343-Bc | 18 | ST-18 complex | 31 | 1143-Bc | 26 | Singleton | |
| 16 | 1193-Bc | 18 | ST-18 complex | 32 | 1162-Bc^#^ | 26 | Singleton | |
| 33 | 1228-Bc^#^ | 26 | Singleton | 51 | 3484-1A-Bc ^#^ | 26 | Singleton | |
| 34 | Y845-Bc^#^ | 26 | Singleton | 52 | 4189-2A-Bc | 26 | Singleton | |
| 35 | 1079-Bc^#^ | 26 | Singleton | 53 | 4189-3A-Bc | 26 | Singleton | |
| 36 | Y1435-Bc | 26 | Singleton | 54 | 810-3A-Bc | 32 | Singleton | |
| 37 | Y1512-Bc | 26 | Singleton | 55 | 1761^-1^C-Bc | 32 | Singleton | |
| 38 | 1736^-3^C-Bc | 26 | Singleton | 56 | Y1810-Bc | 32 | Singleton | |
| 39 | 1761^-2^A-Bc | 26 | Singleton | 57 | 1835^-3^B-Bc | 32 | Singleton | |
| 40 | 1911-Bc | 26 | Singleton | 58 | 1839^-3^B-Bc | 32 | Singleton | |
| 41 | 1935-Bc^#^ | 26 | Singleton | 59 | 2309-1-Bc | 32 | Singleton | |
| 42 | 1938-1-Bc | 26 | Singleton | 60 | 2309-2-Bc | 32 | Singleton | |
| 43 | 2062-1-Bc^#^ | 26 | Singleton | 61 | 2562-Bc | 32 | Singleton | |
| 44 | 2084-Bc^#^ | 26 | Singleton | 62 | 1195-2-Bc | 51 | Singleton | |
| 45 | 2810-3C-Bc^#^ | 26 | Singleton | 63 | 1611-2B-Bc | 72 | ST-111 complex | |
| 46 | 2834-3B-Bc^#^ | 26 | Singleton | 64 | Y1412-Bc | 90 | Singleton | |
| 47 | 2884-2C-Bc^#^ | 26 | Singleton | 65 | 1534-3A-Bc | 90 | Singleton | |
| 48 | 2884-3C-Bc^#^ | 26 | Singleton | 66 | 1536-1B-Bc | 90 | Singleton | |
| 49 | 2889-1B-Bc^#^ | 26 | Singleton | 67 | Y1712-Bc | 90 | Singleton | |
| 50 | 2910-2A-Bc^#^ | 26 | Singleton | 68 | 1912-1-Bc | 90 | Singleton | |
| 69 | 1912-3-Bc | 90 | Singleton | 87 | 1712-1C-Bc | 111 | ST-111 complex | |
| 70 | 2388-Bc | 90 | Singleton | 88 | 237-1-Bc | 127 | Singleton | |
| 71 | 2962-Bc | 90 | Singleton | 89 | 762-2-Bc | 127 | Singleton | |
| 72 | 3536-2A-Bc | 90 | Singleton | 90 | 1785-1A-Bc | 127 | Singleton | |
| 73 | 3610-Bc | 90 | Singleton | 91 | 793-4-Bc | 142 | ST-142 complex | |
| 74 | 39-1-Bc | 92 | Singleton | 92 | Y1589-1-Bc | 142 | ST-142 complex | |
| 75 | 527-1C-Bc | 92 | Singleton | 93 | 3210-1A-Bc | 144 | Singleton | |
| 76 | 743-1-Bc | 92 | Singleton | 94 | 3262-3A-Bc | 144 | Singleton | |
| 77 | 862-Bc | 92 | Singleton | 95 | Y1462-Bc | 156 | ST-18 complex | |
| 78 | 894-1A-Bc | 92 | Singleton | 96 | 1436-2-Bc | 158 | Singleton | |
| 79 | 1012-Bc | 92 | Singleton | 97 | 1535-2A-Bc | 164 | Singleton | |
| 80 | 1437-Bc | 92 | Singleton | 98 | 1537-2C-Bc | 164 | Singleton | |
| 81 | 1635-2B-Bc | 92 | Singleton | 99 | 1939-2-Bc | 164 | Singleton | |
| 82 | 2012-1-Bc | 92 | Singleton | 100 | 2810-1B-Bc | 164 | Singleton | |
| 83 | 3287-2B-Bc | 92 | Singleton | 101 | 3235-1A-Bc | 164 | Singleton | |
| 84 | 4187-1B-Bc | 92 | Singleton | 102 | 995-1-Bc | 164 | Singleton | |
| 85 | Y895-Bc | 92 | Singleton | 103 | 160-1-Bc | 177 | Singleton | |
| 86 | 2210-2-Bc | 104 | Singleton | 104 | 1784-2A-Bc | 177 | Singleton | |
| 105 | 2085-Bc | 177 | Singleton | 123 | 3688-Bc | 205 | ST-205 complex |  |
| 106 | 2193-Bc | 177 | Singleton | 124 | 3835-3A-Bc | 205 | ST-205 complex |  |
| 107 | 2787-Bc | 177 | Singleton | 125 | 3961-2A-Bc | 205 | ST-205 complex |  |
| 108 | 3209-Bc | 177 | Singleton | 126 | 3962-1A-Bc | 205 | ST-205 complex |  |
| 109 | 3784-Bc | 177 | Singleton | 127 | 2909-1A-Bc | 217 | Singleton |  |
| 110 | 2160-Bc | 182 | ST-205 complex | 128 | 1910-4-Bc | 253 | Singleton |  |
| 111 | 2760-1-Bc | 197 | ST-23 complex | 129 | Y442-Bc | 266 | Singleton |  |
| 112 | 4234-1C-Bc | 197 | ST-23 complex | 130 | 1759-1A-Bc | 363 | ST-142 complex |  |
| 113 | 86-Bc | 205 | ST-205 complex | 131 | 544-2B-Bc | 369 | Singleton |  |
| 114 | 711-3-Bc | 205 | ST-205 complex | 132 | Y1639-Bc | 369 | Singleton |  |
| 115 | 2059-1-Bc | 205 | ST-205 complex | 133 | 2338-Bc | 369 | Singleton |  |
| 116 | 2835-3A-Bc^#^ | 205 | ST-205 complex | 134 | 3509-Bc | 369 | Singleton |  |
| 117 | 2884-2B-Bc | 205 | ST-205 complex | 135 | 3139-1B-Bc | 378 | Singleton |  |
| 118 | 2936-2B-Bc | 205 | ST-205 complex | 136 | 2239-Bc | 395 | ST-142 complex |  |
| 119 | 3012-1B-Bc | 205 | ST-205 complex | 137 | 236-Bc | 462 | ST-205 complex |  |
| 120 | 3185-1B-Bc | 205 | ST-205 complex | 138 | Y880-Bc | 462 | ST-205 complex |  |
| 121 | 3539-Bc | 205 | ST-205 complex | 139 | 2535-Bc | 462 | ST-205 complex |  |
| 122 | 3635-Bc^#^ | 205 | ST-205 complex | 140 | 1512-1B-Bc | 465 | ST-18 complex |  |
| 141 | 1712-1A-Bc | 465 | ST-18 complex | 159 | 4234-2B-Bc | 777 | Singleton |  |
| 142 | 2487-1-Bc | 465 | ST-18 complex | 160 | 894-2B-Bc | 811 | Singleton |  |
| 143 | 2238-Bc | 493 | Singleton | 161 | 2710-Bc | 857 | Singleton |  |
| 144 | 62-2-Bc | 770 | Singleton | 162 | 2888-Bc | 869 | ST-205 complex |  |
| 145 | 112-Bc | 770 | Singleton | 163 | 2761-Bc | 959 | ST-18 complex |  |
| 146 | 184-1-Bc | 770 | Singleton | 164 | 1984-2-Bc | 962 | ST-142 complex |  |
| 147 | 209-Bc | 770 | Singleton | 165 | 2010-1-Bc | 962 | ST-142 complex |  |
| 148 | 211-Bc | 770 | Singleton | 166 | 2234-Bc | 990 | Singleton |  |
| 149 | 212-2-Bc | 770 | Singleton | 167 | 3089-1A-Bc | 997 | Singleton |  |
| 150 | 1660-3A-Bc | 770 | Singleton | 168 | 561-3B-Bc | 999 | ST-142 complex |  |
| 151 | 1661-3B-Bc | 770 | Singleton | 169 | 2634-Bc | 1000 | ST-142 complex |  |
| 152 | 1736-1A-2-Bc | 770 | Singleton | 170 | 2061-Bc | 1014 | Singleton |  |
| 153 | 1738-3B-Bc | 770 | Singleton | 171 | 60-Bc | 1017 | ST-205 complex |  |
| 154 | 2010-3-Bc | 770 | Singleton | 172 | 3711-Bc^#^ | 1032 | Singleton |  |
| 155 | 2030-1-Bc | 770 | Singleton | 173 | 4234-1A-Bc | 1050 | Singleton |  |
| 156 | 2262-Bc | 770 | Singleton | 174 | 629-3C-Bc | 1058 | Singleton |  |
| 157 | 2286-Bc | 770 | Singleton | 175 | 2935-2A-Bc | 1065 | ST-205 complex |  |
| 158 | 3838-Bc | 774 | Singleton | 176 | 85-1-Bc | 1066 | ST-205 complex |  |
| 177 | 87-Bc | 1066 | ST-205 complex | 195 | 1585-3A-Bc | 1150 | Singleton |  |
| 178 | 88-Bc | 1066 | ST-205 complex | 196 | 2161-Bc | 1161 | Singleton |  |
| 179 | 239-Bc | 1066 | ST-205 complex | 197 | 1112-Bc | 1168 | Singleton |  |
| 180 | 643-2B-Bc | 1066 | ST-205 complex | 198 | 1160-2-Bc | 1202 | Singleton |  |
| 181 | 2884-1B-Bc | 1066 | ST-205 complex | 199 | Y1860-Bc | 1207 | Singleton |  |
| 182 | 2889-1A-Bc | 1066 | ST-205 complex | 200 | Y879-Bc | 1210 | Singleton |  |
| 183 | 3239-2A-Bc | 1066 | ST-205 complex | 201 | 1109-Bc | 1217 | ST-142 complex |  |
| 184 | 4136-1A-Bc | 1066 | ST-205 complex | 202 | 1359-Bc | 1217 | ST-142 complex |  |
| 185 | 2059-2-Bc | 1084 | ST-205 complex | 203 | 3087-Bc | 1219 | Singleton |  |
| 186 | 2088-1-Bc | 1084 | ST-205 complex | 204 | 3912-Bc | 1223 | Singleton |  |
| 187 | 2088-2-Bc | 1084 | ST-205 complex | 205 | 779-2-Bc | 1237 | ST-142 complex |  |
| 188 | 2960-1A-Bc | 1084 | ST-205 complex | 206 | 792-Bc | 1237 | ST-142 complex |  |
| 189 | 2985-2A-Bc | 1084 | ST-205 complex | 207 | 1809-2C-Bc | 1237 | ST-142 complex |  |
| 190 | 2988-3A-Bc | 1084 | ST-205 complex | 208 | 2043-2-Bc | 1237 | ST-142 complex |  |
| 191 | Y1461-Bc | 1120 | ST-205 complex | 209 | 2559-Bc | 1241 | Singleton |  |
| 192 | 2835-2A-Bc | 1120 | ST-205 complex | 210 | 3461-Bc | 1243 | ST-18 complex |  |
| 193 | 1360-Bc | 1131 | Singleton | 211 | Y293-Bc | 1259 | Singleton |  |
| 194 | 4162-Bc | 1149 | Singleton | 212 | 2111-2-Bc | 1311 | Singleton |  |
| 213 | 3535-Bc | 1311 | Singleton | 231 | 260-1B-Bc | 1431 | ST-142 complex |  |
| 214 | 2938-1B-Bc | 1395 | ST-365 complex | 232 | 2087-1-Bc | 1431 | ST-142 complex |  |
| 215 | 134-Bc | 1417 | ST-205 complex | 233 | 2087-2-Bc^#^ | 1431 | ST-142 complex |  |
| 216 | 136-Bc | 1417 | ST-205 complex | 234 | 3060-2C-Bc | 1431 | ST-142 complex |  |
| 217 | 138-Bc | 1417 | ST-205 complex | 235 | 2362-Bc | 1439 | Singleton |  |
| 218 | 159-Bc | 1417 | ST-205 complex | 236 | 3362-Bc | 1439 | Singleton |  |
| 219 | 160-2-Bc | 1417 | ST-205 complex | 237 | 3639-Bc | 1439 | Singleton |  |
| 220 | 161-Bc | 1417 | ST-205 complex | 238 | 2786-Bc | 1477 | Singleton |  |
| 221 | 184-2-Bc | 1417 | ST-205 complex | 239 | 1610-1A-Bc | 1481 | ST-142 complex |  |
| 222 | 188-1-Bc | 1417 | ST-205 complex | 240 | 2538-2-Bc | 1481 | ST-142 complex |  |
| 223 | 188-2-Bc | 1417 | ST-205 complex | 241 | 2461-Bc | 1483 | Singleton |  |
| 224 | 2178-1-Bc | 1417 | ST-205 complex | 242 | 3538-1A-Bc | 1483 | Singleton |  |
| 225 | 2179-Bc | 1417 | ST-205 complex | 243 | 137-Bc | 1595 | Singleton |  |
| 226 | 62-1-Bc | 1418 | Singleton | 244 | 210-Bc | 1605 | Singleton |  |
| 227 | Y1734-Bc | 1418 | Singleton | 245 | 3135-Bc | 1605 | Singleton |  |
| 228 | 2028-2-Bc | 1418 | Singleton | 246 | 1560-1C-Bc | 1608 | Singleton |  |
| 229 | 2045-Bc | 1418 | Singleton | 247 | 3487-1B-Bc | 1616 | ST-142 complex |  |
| 230 | 2112-2-Bc | 1418 | Singleton | 248 | Y1786-Bc | 1650 | ST-205 complex |  |
| 249 | 1378-Bc | 1681 | Singleton | 267 | 2159-1-Bc | 2178 | ST-205 complex |  |
| 250 | 2610-Bc | 1688 | Singleton | 268 | 2361-Bc | 2188 | Singleton |  |
| 251 | 3761-1C-Bc | 1688 | Singleton | 269 | 2110-1-Bc | 2241* | Singleton |  |
| 252 | 2538-1-Bc | 1712 | Singleton | 270 | 185-Bc | 2259* | ST-18 complex |  |
| 253 | 3909-1C-Bc | 1723 | Singleton | 271 | 187-Bc | 2259* | ST-18 complex |  |
| 254 | 110-Bc | 1784 | Singleton | 272 | 189-Bc | 2259* | ST-18 complex |  |
| 255 | Y815-Bc | 1804 | Singleton | 273 | 186-2-Bc | 2263* | ST-205 complex |  |
| 256 | 2512-1-Bc | 1829 | Singleton | 274 | Y261-Bc | 2268* | Singleton |  |
| 257 | Y562-Bc | 1859 | ST-142 complex | 275 | 279-1C-Bc | 2269* | Singleton |  |
| 258 | 2534-3-Bc | 1859 | ST-142 complex | 276 | 362-1-Bc | 2270* | Singleton |  |
| 259 | 2136-Bc | 1866 | Singleton | 277 | Y362-Bc | 2271* | Singleton |  |
| 260 | 3110-3B-Bc | 1892 | Singleton | 278 | 379-3C-Bc | 2272* | Singleton |  |
| 261 | 4012-2A-Bc | 1892 | Singleton | 279 | Y642-Bc | 2273* | Singleton |  |
| 262 | 1910-2-Bc | 1929 | Singleton | 280 | 1128-Bc | 2274* | Singleton |  |
| 263 | 3709-1A-Bc^#^ | 1947 | ST-18 complex | 281 | 1144-Bc | 2275* | Singleton |  |
| 264 | 2111-1-Bc^#^ | 2150* | ST-142 complex | 282 | 1187-Bc | 2276* | Singleton |  |
| 265 | 3536-1B-Bc | 2150* | ST-142 complex | 283 | 1229-Bc | 2277* | Singleton |  |
| 266 | 3611-2B-Bc | 2150* | ST-142 complex | 284 | 1279-Bc | 2278* | Singleton |  |
| 285 | 1294-Bc | 2279* | Singleton | 303 | 1836-2B-Bc | 2297* | Singleton |  |
| 286 | 1394-Bc | 2280* | Singleton | 304 | Y1838-Bc | 2298* | ST-18 complex |  |
| 287 | Y860-Bc | 2281* | ST-205 complex | 305 | Y1861-Bc | 2299* | Singleton |  |
| 288 | 980-Bc | 2282* | Singleton | 306 | 1884-1A-Bc | 2300* | Singleton |  |
| 289 | 1436-1C-Bc | 2283* | Singleton | 307 | 1886-1-Bc | 2301* | Singleton |  |
| 290 | Y1439-Bc | 2284* | ST-205 complex | 308 | 1886-2-Bc | 2302* | Singleton |  |
| 291 | Y1460-Bc | 2285* | Singleton | 309 | 1887-1C-Bc | 2303* | Singleton |  |
| 292 | Y1484-Bc | 2286* | Singleton | 310 | 1889-2C-Bc | 2304* | Singleton |  |
| 293 | 1484-3B-Bc | 2286* | Singleton | 311 | 1959-Bc | 2305* | Singleton |  |
| 294 | 1586-3B-Bc | 2288* | Singleton | 312 | 1986-3-Bc | 2306* | Singleton |  |
| 295 | 1612-1A-Bc | 2289* | Singleton | 313 | 2010-2-Bc | 2307* | Singleton |  |
| 296 | Y1612-Bc | 2290* | Singleton | 314 | 2044-2-Bc | 2308* | Singleton |  |
| 297 | 1634-1A-Bc | 2291* | Singleton | 315 | 2086-2-Bc | 2309* | ST-23 complex |  |
| 298 | 1661-2-Bc | 2292* | Singleton | 316 | 2110-2-Bc | 2310* | Singleton |  |
| 299 | 1686-1C-Bc | 2293* | ST-142 complex | 317 | 2112-1-Bc | 2311* | Singleton |  |
| 300 | 1711-1A-Bc^#^ | 2294* | ST-205 complex | 318 | 2112-3-Bc | 2312* | Singleton |  |
| 301 | 1761-3B-Bc | 2295* | Singleton | 319 | 2210-1-Bc | 2313* | Singleton |  |
| 302 | 1810-2B-Bc | 2296* | Singleton | 320 | 2237-Bc | 2314* | Singleton |  |
| 321 | 2288-Bc | 2315* | ST-205 complex | 339 | 2910-3A-Bc | 2332* | ST-142 complex |  |
| 322 | 2336-1-Bc | 2316* | Singleton | 340 | 2960-2C-Bc | 2333* | ST-205 complex |  |
| 323 | 2360-Bc | 2317* | Singleton | 341 | 2985-1C-Bc | 2334* | ST-365 complex |  |
| 324 | 2437-Bc | 2318* | Singleton | 342 | 3110-2B-Bc | 2335* | ST-111 complex |  |
| 325 | 2439-Bc | 2318* | Singleton | 343 | 3210-2B-Bc | 2336* | Singleton |  |
| 326 | 2460-1-Bc | 2319* | Singleton | 344 | 3239-Bc | 2337* | ST-18 complex |  |
| 327 | 2462-Bc | 2320* | Singleton | 345 | 3239-1C-Bc | 2338* | ST-111 complex |  |
| 328 | 2484-Bc | 2321* | Singleton | 346 | 3239-3A-Bc | 2339* | ST-205 complex |  |
| 329 | 2485-Bc | 2322* | Singleton | 347 | 3259-1B-Bc | 2340* | ST-23 complex |  |
| 330 | 2487-4-Bc | 2323* | Singleton | 348 | 3260-Bc | 2341* | Singleton |  |
| 331 | 2510-1-Bc | 2324* | Singleton | 349 | 3311-2A-Bc | 2342* | ST-205 complex |  |
| 332 | 2512-2-Bc | 2325* | Singleton | 350 | 3334-1A-Bc | 2343* | ST-142 complex |  |
| 333 | 2534-2-Bc | 2326* | Singleton | 351 | 3762-Bc | 2343* | ST-142 complex |  |
| 334 | 2585-Bc | 2327* | Singleton | 352 | 3436-1B-Bc | 2344* | ST-205 complex |  |
| 335 | 2760-2-Bc | 2328* | Singleton | 353 | 3462-Bc | 2345* | ST-142 complex |  |
| 336 | 2884-1C-Bc | 2329* | Singleton | 354 | 3462-1A-Bc | 2346* | Singleton |  |
| 337 | 2884-3A-Bc | 2330* | Singleton | 355 | 3539-1C-Bc | 2347* | Singleton |  |
| 338 | 2910-1A-Bc | 2331* | ST-205 complex | 356 | 3611-3C-Bc | 2347* | Singleton |  |
| 357 | 4185-1A-Bc | 2347* | Singleton | 363 | 3984-1A-Bc | 2354* | ST-205 complex |  |
| 358 | 4185-2A-Bc | 2347* | Singleton | 364 | 4062-Bc | 2355* | Singleton |  |
| 359 | 3560-1A-Bc | 2348* | ST-142 complex | 365 | 4211-Bc | 2358* | Singleton |  |
| 360 | 3638-Bc | 2350* | Singleton | 366 | 4234-3A-Bc | 2359* | ST-23 complex |  |
| 361 | 3760-Bc | 2351* | Singleton | 367 | 4256-Bc | 2360* | ST-365 complex |  |
| 362 | 3837-Bc | 2353* | Singleton | 368 | 429-2A-Bc | 2361* | ST-205 complex |  |

* represents the new ST; # represents potential emetic strains
